# A practical guide for assessing respiratory burst and phagocytic cell activity in the fathead minnow, an emerging model for immunotoxicity

**DOI:** 10.1016/j.mex.2020.100992

**Published:** 2020-07-10

**Authors:** Leah M. Thornton Hampton, Marlo K. Sellin Jeffries, Barney J. Venables

**Affiliations:** a Department of Biological Sciences, University of North Texas, Denton, TX, USA; b Department of Biology, Texas Christian University, Fort Worth, TX, USA

**Keywords:** Kidney cell extraction, Phagocytosis, Respiratory burst, Cellular immune function, Fish

## Abstract

Measures of respiratory burst and phagocytic cell activity are frequently utilized to assess cellular immune function in teleosts. Respiratory burst predominately occurs in neutrophils and causes the release of reactive oxygen species to kill pathogens. Phagocytosis is the process by which pathogens are engulfed and destroyed by various immune cells. Though a variety of approaches have been utilized to measure respiratory burst and phagocytic cell activity, assays that rely only on common laboratory equipment (e.g., plate reader) may offer advantages over those that rely on more specialized equipment (e.g., flow cytometer). The goal of the current study was to optimize and validate the use of a colorimetric plate-based respiratory burst and fluorometric plate-based phagocytic cell activity assays for use with kidney cells from the fathead minnow (*Pimephales promelas*), an emerging immunotoxicity model. In addition, a protocol for the dissection of kidney tissue followed by the extraction of kidney cells, as well as recommendations and resources for future experiments utilizing each of these assays, are provided.

• All methods are optimized for use with the fathead minnow or similar teleost species.
• Respiratory burst and phagocytic cell activity are measured using a standard plate reader.

## Graphical abstract

**Figure:**
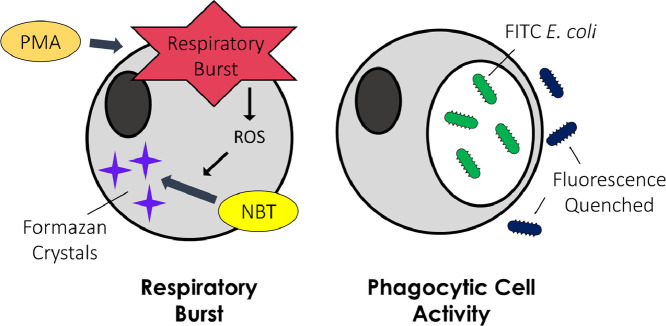

Specifications table

**Table:** 

Subject Area	Agricultural and biological sciences
More specific subject area	Immunotoxicology
Method name	• Dissection and cell extraction from fathead minnow kidney tissue • Assessment of respiratory burst in fathead minnow kidney cells • Assessment of phagocytic cell activity in fathead minnow kidney cells
Name and reference of original method	• C.J. Secombes. Isolation of salmonid macrophages and analysis of their killing activity. In: Techniques in Fish Immunology. (1990), pp. 139–154. • J. Ninković, S. Roy. High throughput fluorometric technique for assessment of macrophage phagocytosis and actin polymerization. J. Vis. Exp. 93, (2014).
Resource availability	NA

## Method details

### Respiratory burst

Respiratory burst is characterized by the rapid release of reactive oxygen species, predominately from neutrophils, for pathogen killing. As a key defense mechanism against a wide range of pathogens, the assessment of respiratory burst is a popular endpoint in immunological studies conducted with teleosts [1]. Respiratory burst is often assessed via flow cytometry. However, in cases where only low numbers of cells may be harvested, or a flow cytometer is unavailable, alternative methods may be required. In these instances, plate-based colorimetric assays provide a relatively simple, cost-effective approach for estimating respiratory burst *ex vivo* via the production of superoxide [2]. In this assay, which may be applied to any cell type capable of respiratory burst, respiratory burst may be induced via one of several mitogens (e.g., phorbol 12-myristate 13-acetate (PMA), lipopolysaccharide (LPS), N-formyl-methionyl-leucyl-phenylalanine (FMLP)) [3]. Alongside of the mitogen, nitroblue tetrazolium (NBT), is added to isolated cell suspensions. The NBT, which is yellow in color, diffuses into the cells where it is reduced by reactive oxygen species, particularly superoxide, to produce formazan crystals, which upon dissolution in potassium hydroxide (KOH) and dimethyl sulfoxide (DMSO) produce a blue color. This dramatic color change can be easily measured with a standard plate reader and because the absorbance of the solution is directly related to the amount of superoxide produced, absorbance can be used to estimate respiratory burst activity. In addition, a set of reactions is carried out in the presence of superoxide dismutase (SOD), which catalyzes the conversion of superoxide (but not other free radicals) to oxygen and hydrogen peroxide, to demonstrate assay specificity for superoxide [3,4].

The goal of this study was to adapt, optimize and validate the NBT assay (as described by [4] and [3]) for use with kidney cells harvested from the fathead minnow (*Pimephales promelas*). To our knowledge, this assay has not yet been utilized to assess respiratory burst in this species, a common model for assessing general toxicity and emerging model in the field of immunotoxicity [5], [6], [7], [8], [9], [10], [11], [12], [13]. This study explored the following experimental parameters to maximize the performance of the respiratory burst assay: 1) PMA concentration, 2) incubation time, 3) erythrocyte lysis, and 4) presence of superoxide dismutase (SOD). Optimization efforts included determination of appropriate PMA concentrations and incubation times given that both have potential effects on the overall magnitude of the respiratory burst response. Erythrocyte lysis was investigated as it affects the relative proportion of leukocytes, the cell type predominately responsible for respiratory burst, in each reaction. The effect of SOD was tested given its frequent inclusion as an indicator of reaction specificity.

### Phagocytic cell activity

Phagocytosis is the internalization and subsequent destruction of foreign material or microbes, such as bacterial pathogens. This essential component of immune defense may be performed by a wide variety of cell types. In teleosts, phagocytosis has been observed in macrophages, granulocytes, dendritic cells, lymphocytes and thrombocytes [14]. Phagocytosis is commonly assessed via microscopy or flow cytometry; each having unique advantages and disadvantages. Microscopy can be time consuming and laborious, but it allows for clear discernment between engulfed particles and those associated with the cell surface. In contrast, flow cytometry is relatively rapid; however, intra- and extracellular particles are often indistinguishable from one another [15]. A fluorometric plate-based approach provides a simple and rapid alternative by which to assess general phagocytic cell function *ex vivo* while also allowing for differentiation between intra- and extracellular particles. In this assay, cell suspensions are incubated with fluorescently-labeled, heat-killed bacteria, which are rapidly engulfed by phagocytes. Immediately prior to measurement, trypan blue is added to quench extracellular fluorescence and the fluorescence of phagocytosed particles is quantified utilizing a standard plate reader. Increases in fluorescence intensity are indicative of phagocytic cell activity [16].

This study sought to adapt, optimize and validate the methods originally outlined by Ninković & Roy [16] for use with murine macrophage cell lines for use with fathead minnow kidney cells. To maximize the performance of this assay, the experimental parameter of erythrocyte lysis was explored. The lysis and removal of erythrocytes was hypothesized to increase the proportion of leukocytes, including phagocytes, in each reaction relative to other cell types, thus enhancing phagocytic cell activity.

### Protocols

#### Materials

##### Dissection and cell isolation

Freshly-prepared tricaine mesylate (MS-222) (0.3 g/L – lethal dose) buffered sodium bicarbonate to ensure a pH consistent with that of the water that fish have been maintained in

General dissection supplies (e.g., forceps, scissors, pins, scalpel, dissection pan)

Heparinized capillary tubes

70% EtOH (preferably in spray bottle)

100 – 250 mL beaker for 70% EtOH

Paper towels

Microscale

Sterile 1.5 mL Eppendorf tubes

Sterile cell media (supplemented Leibovitz's L-15 cell media (catalog #L5520, Sigma Aldrich)); Fetal Bovine Serum 5% (catalog #F4135, Sigma Aldrich), Penicillin/Streptomycin 1% (catalog #P4333, Sigma Aldrich), 1.5 M HEPES 1% (catalog #H4034, Sigma Aldrich), L-Glutamine 0.5% (catalog #G7513, Sigma Aldrich)

– stored at 4 °C

Cooler filled with ice

Sterile 1 mL syringes

Paper clip

Sterile glass wool

Plastic pestles

Sterile 5 mL screw cap tubes

Centrifuge with adaptors for 5 mL tubes

Trypan blue (0.4%) (catalog #76,180–676, VWR)

Hemocytometer

Microscope

Tissue Culture Treated 96 Well Flat Bottom Plate (part #667,196, Dot Scientific Inc.)

Set of micropipettes and sterile tips

Laminar flow hood

Incubator set at 30 °C (CO_2_ not required due to use of l-15 media)

*Respiratory Burst*

Dissecting microscope

Nitroblue tetrazolium (NBT) (catalog #N6639, Sigma Aldrich) Stock Solution (2.5 mg/mL) prepared in Hank's Balanced Salt

Solution (HBSS) – To be prepared fresh for each assay

Bovine superoxide dismutase (SOD) (catalog #S5395, Sigma Aldrich) (Stock Solution (300 µg/mL) prepared in HBSS

– stored at −20 °C
– Note: Consider the use of membrane-permeable SOD; see section titled, Respiratory Burst: Erythrocyte Lysis and Superoxide Dismutase

Phorbol 12-myristate 13-acetate (PMA) (catalog #76,102–732, VWR) 1 mg/mL stock solution prepared in DMSO; Working Solution (100 µg/mL) prepared in HBSS

– stored at −20 °C
– light sensitive

Sterile cell media (same as above)

70% MeOH

2 M KOH

Dimethyl sulfoxide (DMSO)

Reagent reservoirs

Multichannel pipette

Set of micropipettes and sterile tips

Standard plate reader (absorbance)

Laminar flow hood

Incubator set at 30 °C

*Phagocytic Cell Activity*

Dissecting microscope

Fluorescein (FITC) conjugated *Escherichia coli* K-12 BioParticles (0.5 mg/mL) prepared in HBSS and 2 mM sodium azide (catalog #E2861, ThermoFisher Scientific)

– stored at 4 °C
– protected from light

Vortex

Trypan blue (0.4%) (catalog #76,180–676, VWR)

Aluminum foil

Set of micropipettes and sterile tips

Centrifuge for 96 well plates

Standard plate reader (fluorescence)

Incubator set at 30 °C

Laminar flow hood

##### Preparation of materials

Note: Complete the following under sterile conditions in a laminar flow hood.

1. Loosely pack syringes with sterilized glass wool using forceps and unwound paper clip previously doused in 70% EtOH.
2. Pipette 100 µL of previously prepared cell media into sterile 1.5 mL Eppendorf tubes. Fill one tube per sample. Label tubes according to sample number and place on ice outside hood.
3. Label 2 sets of sterile 5 mL screw cap tubes according to sample number.
4. Pipette 90 µL of cell media into 1 set of 1.5 mL Eppendorf tubes and label according to sample number and “1:10”.

##### Kidney tissue collection

Note: If possible, conduct dissections under sterile conditions in a laminar flow hood. Dissections may be completed outside of a laminar flow hood, but care must be taken to work quickly and decisively to minimize the risk of contamination of tissues and subsequent cell suspensions.

**Fig. 1:**
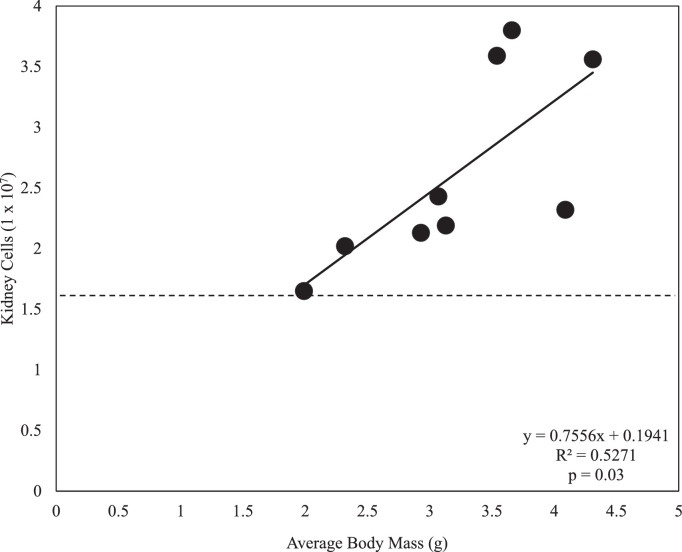
Regression analysis between total body mass and cell yield (*n* = 9) of adult male fathead minnows. Data in panel may be used to predict kidney cell yield from body mass. Dashed lines represents minimum cell yield required to perform both respiratory burst and phagocytic cell assays as described.

1. Euthanize fish via immersion in a lethal dose of buffered MS-222 (0.3 g/L).
2. Following the cessation of operculum movement (<3 min for adults), remove fish from MS-222 solution, gently dry on a paper towel and measure total mass.
3. Immediately sever the caudal fin using a scalpel and collect blood in a heparinized capillary tube. Remove as much blood as possible to reduce the numbers of peripheral erythrocytes in the final cell suspension.
4. Use a spray bottle to douse the outside of the fish with 70% EtOH. Dip dissection tools in 70% EtOH and dab dry on a clean paper towel. Begin the dissection normally, taking care not to puncture the intestines. It is recommended to remove viscera up and out to the side of the fish to allow for the easy removal of the kidney tissue.
5. Immediately before removing the kidney tissue, place the Eppendorf tube previously filled with 100 µL of cell media on the microscale and tare. Dip forceps in 70% EtOH and completely dry on a clean paper towel or Kim wipe. Residual ethanol on the forceps will desiccate cells. Remove all kidney tissue from the body cavity and place into pre-tared Eppendorf tube and return to scale. Record kidney tissue mass.
6. Clean dissection pan and dissection tools with 70% EtOH and repeat steps 1–5 for each fish, pooling the appropriate number of tissues together per Eppendorf tube. Return Eppendorf tubes to ice after placing tissues in tube. To determine the number of fish to pool per sample for the desired number of cells for subsequent assays, refer to Fig. 1.

##### Creation of cell suspension

Note: Complete the following under sterile conditions in a laminar flow hood.

1. Once all tissues have been dissected, return to the laminar flow hood and gently homogenize each sample with a sterile plastic pestle. Use a new plastic pestle for each sample.
2. Add 1000 µL of cell media to each sample, rinsing the plastic pestle over the tube in the process. Gently pipette the homogenate up and down ~10–15 times using a 100–1000 µL pipette.
3. To remove remaining large pieces of tissue, remove the plunger and cap of a syringe previously packed with glass wool and hold the syringe over the previously labeled 5 mL screw cap tube in case of dripping. Pipette the homogenate into the syringe until full. Gently push the plunger back into the syringe to gently filter the homogenate into the 5 mL screw cap tube.
4. After all samples have been filtered, centrifuge samples for 10 min at ~180 g at room temperature.
5. Remove and dispose of supernatant being careful not to disturb the cell pellet at the bottom of the tube. Add 1 mL of fresh cell media (room temperature), gently pipetting up and down to resuspend the pellet.
6. Repeat steps 4 and 5 for a total of 2 washes.
7. After washing the cells twice, resuspend the cells in 1 mL of fresh media. Gently pipette up and down ~10–15 times to mix. Immediately transfer (cells settle quickly!) 10 µL of cell suspension to the Eppendorf tube containing 90 µL of media and labeled “1:10”.
8. Return all solutions containing cells to ice.

##### Determination of cell viability and concentration via hemocytometer

Note: Steps 1 and 2 may be performed outside of the laminar flow hood. All other steps should be performed inside a laminar flow hood.

1. For each sample, use a 20–200 µL pipette to gently mix the 1:10 dilution of each cell suspension and transfer 10 µL to a new 1.5 mL Eppendorf tube containing 10 µL of trypan blue and mix by gently pipetting up and down. Apply 10 µL of the mixture to the hemocytometer and allow cells to settle for ~30–60 s.
2. Count live and dead cells in 1–4 squares on the hemocytometer grid (Fig. 2). Average counts together and multiply by the dilution factor (10, if performing a 1:10 dilution), the dilution factor of trypan blue (2) and then by 10^4^ to determine the concentration of cells (ex. mean number of cells in one square x dilution factor x dilution factor of trypan blue x 10^4^ = concentration of cell suspension (cells/mL)). Determine the percentage of live and dead cells to ensure adequate viability (≥ 80%).

**Fig. 2:**
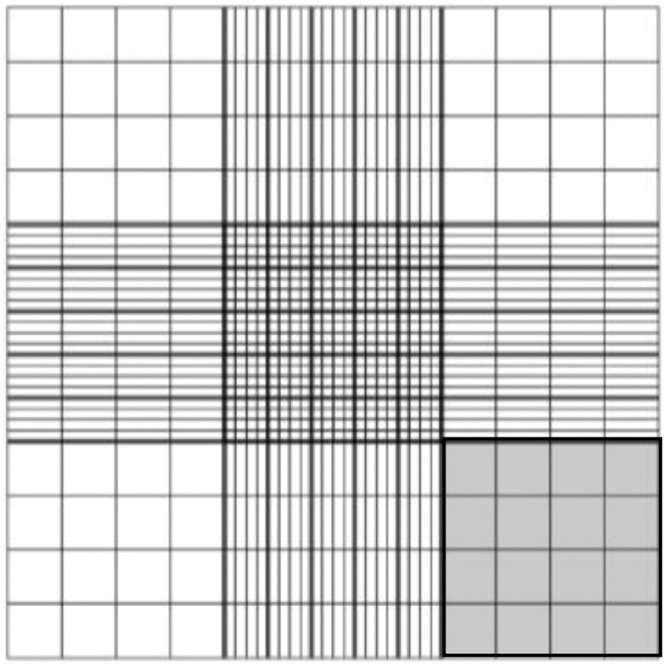
Example of hemocytometer grid. The shaded area indicates one “square.”.
3. Adjust the number of live cells to the required concentration for the desired assay by adding the appropriate volume of cell media. Refer to the Cell Suspension Dilution Sheet in the Supplemental Materials for calculating dilutions. For superoxide production and phagocytic activity, cells should be adjusted to 6 × 10^6^ cells/mL and 100 µL is added to each well. A minimum volume of 2600 µL is required per sample to conduct both assays (1300 µL each). When filling wells, be sure to mix cell suspensions often via gentle pipetting. Include blank wells for each treatment with only cell media and no cell suspension. Refer to the Respiratory Burst Plate or Phagocytic Cell Activity Plate in the Supplemental Materials for examples of suggested plate set ups.
4. After cells have been plated, place the plate in a 30 °C humidified incubator to recover overnight. The injection of CO_2_ is unnecessary due to the use of Leibovitz's L-15 cell media.

##### Respiratory burst

Note: Complete the following under sterile conditions in a laminar flow hood.

1. After allowing the cells to recover overnight, prepare the following reaction mixes according to Table 1. Refer to the Respiratory Burst Reaction Mix Sheet in the Supplemental Materials for calculating volumes of each component.

**Table 1:** Stock solution and final well concentrations of superoxide production reaction mix reagents. The symbol “+” indicates the presence of the regent in the given reaction mix. Refer to the Superoxide Reaction Mix Sheet in the Supplemental Materials for calculating volumes of each component. Note: Nitroblue tetrazolium stock solution is to be made fresh for each assay.

Reagent	Stock Solution Concentration	Final Well Concentration	Reaction Mix 1	Reaction Mix 2	Reaction Mix 3	Reaction Mix 4
Nitroblue Tetrazolium (NBT)	2.5 mg/mL	0.8 mg/mL	+	+	+	+
phorbol 12-myristate 13-acetate (PMA)	100 µg/mL	0.5 µg/mL			+	+
Superoxide Dismutase (SOD)	300 µg/mL	30 µg/mL		+		+
2. Gently remove cells from the incubator and observe under a microscope to check the health of the cells. There should be no signs of contamination or cell death.
3. Using a single-channel or multichannel pipette, remove 50 µL of media from the top of each well. Cells should have settled to the bottom and therefore not be removed from the plate, but if you are at all uncomfortable, it is suggested that a single-channel pipette is used.
4. After vigorously vortexing, add 50 µL of each reaction mixture in triplicate to each sample. Each sample should receive each reaction mix in triplicate. See Respiratory Burst Plate for further clarification.
5. Immediately after adding the appropriate reaction mixes, return the plate to the incubator for 1 h
6. Following incubation, use a multichannel pipette and a reagent reservoir to add 100 µL 70% MeOH to each well.
7. Continuing to use a multichannel pipette (but changing pipette tips each time), immediately remove all solution from the wells and wash each well with an additional 100 µL 70% MeOH two more times. After the second wash, remove any remaining solution and allow the plate to air dry at room temperature. Place the plate under a sample box lid without the plate lid on to avoid contaminants falling into the wells.
8. After the plate is completely dry (~20–30 min), use a multichannel pipette to add 120 µL of 2 M KOH to each well followed by 140 µL of DMSO and mix thoroughly by pipetting up and down (~10–15 times) to dissolve formazan.
9. Immediately measure the absorbance of the solution on a standard plate reader at 620 nm. Subtract mean values for blank wells from mean values of experimental wells receiving the same reaction mix.

##### Phagocytic cell activity

Note: Complete the following under sterile conditions in a laminar flow hood.

1. Gently remove cells from the incubator and observe under a microscope to check the health of the cells. There should be no signs of contamination or cell death.

Note: Plan ahead in the following steps to reduce the time cells are outside of the incubator as much as possible. Phagocytosis occurs rapidly. Therefore, any preliminary steps taken to increase efficiency while taking fluorescence measurements will be beneficial (i.e., plate reader is on and correct acquisition protocol has been previously programmed).

2. Acquire the FITC-conjugated *E. coli* bioparticles (0.5 mg/mL), vortex and add 40 µL to all sample and blank wells. This equates to a 1:10 cell to particle ratio based on manufacturer specifications for the number of particles per mg.
3. Immediately add 50 µL to the 0 h timepoint wells and centrifuge the plate at 500 rpm for 5 min to bring particles to the bottom of each well and facilitate particle-to-cell interaction.
4. Immediately measure the fluorescence intensity of the wells corresponding to the 0 h timepoint using the standard plate reader (excitation filter: λ = 488 nm, emission filter: λ = 518 nm).
5. Return the plate to the incubator and begin the timer for the next timepoint. Loosely cover plate in foil to avoid exposure to light.
6. At each timepoint, remove the plate from the incubator, add 50 µL of trypan blue to the wells corresponding to the given timepoint and measure the fluorescence intensity as previously described. There is no need to centrifuge the plate each time. Repeat until all chosen timepoints have been completed.
7. To determine the amount of phagocytic cell activity over time, subtract the mean fluorescence intensity values at 0 h from the mean fluorescence intensity values at each subsequent timepoint to eliminate background fluorescence in each sample.

## Method optimization

### Animal husbandry

All experimental procedures involving fathead minnows were approved by the TCU Institutional Animal Care and Use Committee (protocol # 17/13). Adult fathead minnows utilized in this experiment were generated in-house in the Texas Christian University (TCU) aquatic facility. Minnows (~11 months post hatch) were housed in 75 L tanks and maintained at 26 °C with a 16 h light:8 h dark photoperiod. All fish were fed commercially available flake food (Tetramin) twice daily ad libitum. Uneaten food and waste were removed during daily one-third water changes.

### Respiratory burst: cell washing

Previous iterations of this assay have described the washing of cells with HBSS immediately prior to the addition of the reaction mix containing NBT, PMA and/or SOD. However, it was found that fathead minnow kidney cells were only weakly adherent to tissue-treated culture plates, and that washing with HBSS, no matter how gently, resulted in a substantial loss of cells from wells. Cell loss caused high variation between technical replicates, and the mean absorbance values for experimental wells were often less than that of blanks (below the detection limit). To remediate this issue, cells were left undisturbed following plating in a total volume of 100 µL, and only 50 µL were removed from the top of the well immediately prior to the addition of reaction mixes to prevent the loss of cells. When utilizing this approach, additional reagents are not consumed by accounting for changes in reaction mix volumes.

### Respiratory burst: PMA concentration & incubation time

To determine the optimal PMA concentration and incubation time post stimulus, kidney cells were harvested from nine adult male fathead minnows and pooled into groups of three (*n* = 3) according to the previously outlined methods. Following plating and overnight recovery, 50 µL of media was removed from the top of each well. Cells were incubated for 0, 30, 60 or 120 min in a 30 °C humidified incubator following the addition of PMA at concentrations of 0, 0.5, 1 or 2 µg/mL and NBT concentrations of 0.8 mg/mL. The total volume in each well was restored to 100 µL with media. At the end of each incubation period, plates were processed as described previously. Each reaction was conducted in triplicate and mean values for blank wells (no cells) were subtracted from mean experimental well values.

**Fig. 3:**
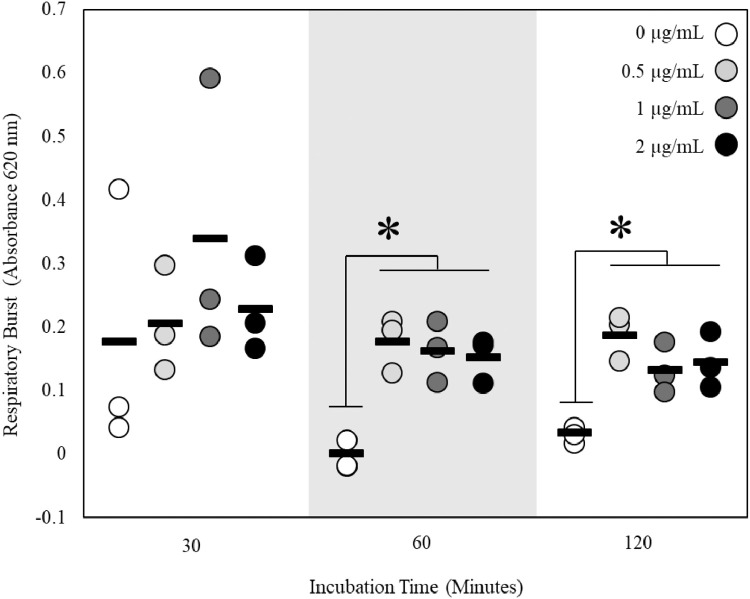
Determination of optimum incubation time and phorbol 12-myristate 13-acetate (PMA) dose (as indicated by shading) for respiratory burst in adult fathead minnow kidney cells (*n* = 3). Bars indicate mean. Asterisks indicate significant differences between doses within a timepoint.

Initially, data from this experiment were analyzed using a two-way ANOVA with incubation time and PMA concentration as the factors. Because there was no significant effect of the interaction between these factors (*p* = 0.66), differences between concentrations at the same timepoint and differences between timepoints at the same concentration were determined via an analysis of variance (ANOVA) followed by a Tukey's post-hoc test. In cases of unequal variance, a Kruskal Wallis test was used. For this and all subsequent analyses, the statistical software package JMP 11.0 was used and statistical significance was set at α = 0.05. When incubated in the absence of PMA, there were no significant differences in the respiratory burst activity of cells incubated for 30, 60 or 120 min (Fig. 3, Kruskal Wallis, *p* = 0.06). This lack of difference in respiratory burst activity as a function of incubation time was also noted when cells were incubated with 0.5, 1 or 2 µg/mL PMA (ANOVA or Kruskal Wallis, all p values ≥ 0.09). When the impact of PMA concentration on assay performance was examined, no differences in respiratory burst were detected between PMA concentrations after 30 min of incubation (ANOVA, p value = 0.64). However, PMA concentration significantly impacted respiratory burst measured at 60 and 120 min (ANOVA, all p values < 0.01). Specifically, respiratory burst was significantly elevated in cells treated with 0.5, 1 and 2 µg/mL PMA relative to those that were untreated with PMA.

An incubation time of 60 min and a PMA concentration of 0.5 µg/mL were chosen as the optimal experimental conditions for this assay. Though incubation time itself did not impact respiratory burst at any of the PMA concentrations tested, there was high variation between replicates when an incubation time of 30 min was utilized. As such, this incubation time is not recommended as statistical differences in respiratory burst activity between samples would likely be difficult to detect without large sample sizes. Furthermore, neither increases in PMA concentration nor incubation time increased formazan production.

### Respiratory burst: erythrocyte lysis & superoxide dismutase

To determine the influence of SOD and erythrocyte lysis, kidney cells were harvested from six adult male fathead minnows and pooled into groups of two (*n* = 3) according to the previously outlined methods. Immediately following filtration, half of each cell suspension was treated with 2 mL of ammonium chloride buffer (9:1 solution 0.16 M NH_4_Cl:0.17 M Tris, pH = 7.65) and rocked gently at room temperature for ~5 min to lyse erythrocytes. The remaining half of each cell suspension remained untreated. Following plating and overnight recovery, 50 µL of media was gently removed from the top of each well to avoid disturbing loosely attached cells. Cells were then incubated with or without 30 µg/mL SOD. All cells were incubated for 60 min in a 30 °C humidified incubator following the addition of 0.5 µg/mL PMA and 0.8 mg/mL NBT. Following the incubation period, plates were processed as described previously. All reactions were carried out in triplicate and mean values for blank wells (no cells) were subtracted from mean experimental well values.

**Fig. 4:**
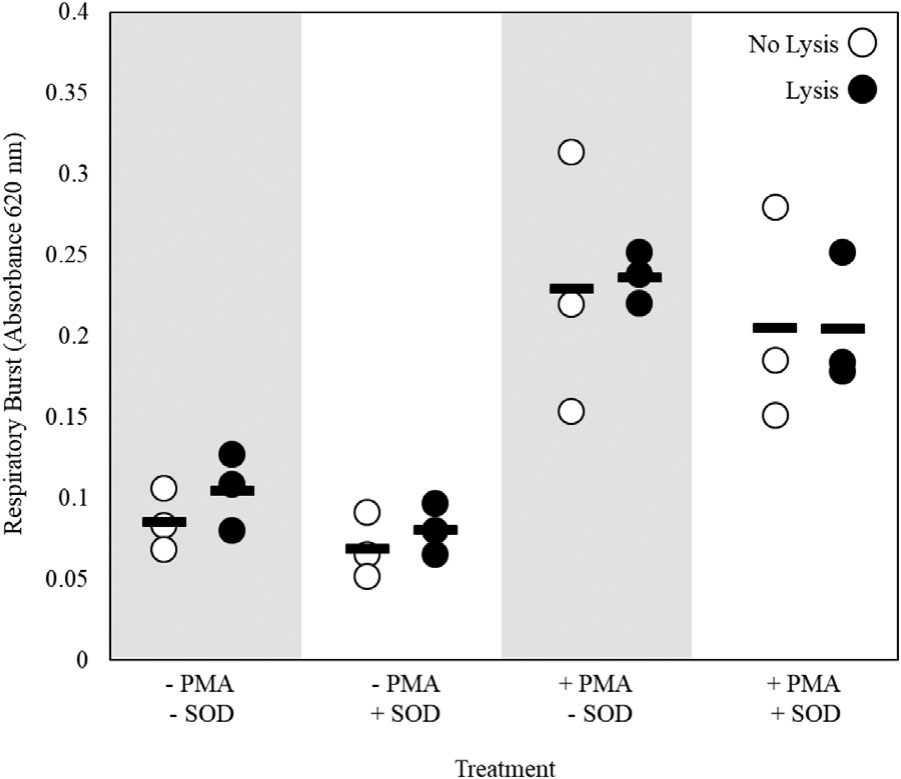
Determination of optimum conditions for the presence of 30 µg/mL superoxide dismutase (SOD) and erythrocyte lysis for respiratory burst in unstimulated or stimulated adult fathead minnow kidney cells (*n* = 3). Stimulated cells were treated with 0.5 µg/mL phorbol 12-myristate 13-acetate (PMA). Bars indicate mean.

Initially, data from this experiment were analyzed using a two-way ANOVA with SOD presence and erythrocyte lysis as the factors. Because there was no significant effect of the interaction between these factors with or without the addition of PMA (all p values ≥ 0.74), differences between untreated and erythrocyte-lysed cell suspensions were compared within SOD and PMA treatment using a t-test. Differences between cells incubated with or without the presence of SOD were compared within lysis and PMA treatment also using a t-test. No significant differences were detected for any of these comparisons (Fig. 4, t-test, all p values ≥ 0.22).

For this assay, it was determined that the lysis of erythrocytes does not affect assay performance and is therefore unnecessary. Furthermore, the lysis of erythrocytes reduces the total cell yield, possibly limiting sample sizes and the number of cellular assays which can be performed using the same cell suspension.

The addition of SOD did not induce any statistically-significant changes in the absorbance measured. Many previous studies have included SOD to demonstrate the specificity of the reaction [3,^4^,[17], [18], [19], [20], [21] as SOD catalyzes the reaction between water and superoxide to yield oxygen and hydrogen peroxide, thus consuming superoxide and preventing the reduction of NBT [3,22]. However, previous studies have only been able to achieve up to ~40–50% inhibition by incubating cells with exogenous SOD [3,^17^,^22^,23], and only ~10–14% inhibition was achieved in the present study. Even when the concentration of SOD was increased, there was no indication of increased inhibition as the mean (± standard deviation) absorbance of stimulated cells incubated with 30 and 60 µg/mL was 0.32 ± 0.03 and 0.33 ± 0.01, respectively. This lack of inhibition is likely because SOD has limited cell permeability and is unable to infiltrate the cells at concentrations high enough to completely inhibit NBT reduction inside the cell [3,22]. Another possibility is that superoxide is only partially contributing to the reduction of NBT as SOD only scavenges superoxide and no other reactive oxygen species. Specifically, additional reactive oxygen species such as nitric oxide (NO) may be produced by respiratory burst and contribute to NBT reduction. Experiments by Choi et al. [3] demonstrated that NO production was not significantly induced at 30 min or 20 h following stimulation with PMA, suggesting that NO does not contribute to formazan production when respiratory burst is induced with PMA. Yet, this was only tested in a single cell line (RAW 264.7) under a specific set of experimental conditions. While the reduction of NBT and subsequent production of formazan crystals is most likely a result of superoxide anion production, the contribution of another free radical(s) cannot be ruled out entirely. Because the SOD used here was not specifically designed to be membrane permeable, it is not possible to tease apart the relative contributions of intracellular superoxide versus other free radicals to overall respiratory burst activity. As such, to definitively confirm that NBT reduction is due exclusively to superoxide anion, it is recommended that a membrane permeable form of SOD be utilized.

### Phagocytic cell activity: erythrocyte lysis

To determine the effect of erythrocyte lysis on phagocytic cell activity, kidney cells were harvested from 12 adult fathead minnows and pooled into groups of four (*n* = 3). Erythrocytes were lysed as described previously. Following plating and overnight incubation, FITC-labeled E. coli particles were added to all wells and fluorescence intensity was measured at 0, 1, 2 and 4 h. All reactions were conducted in triplicate and the value for mean fluorescence intensity at 0 h was subtracted from measurements made at all subsequent timepoints to account for background fluorescence.

**Fig. 5:**
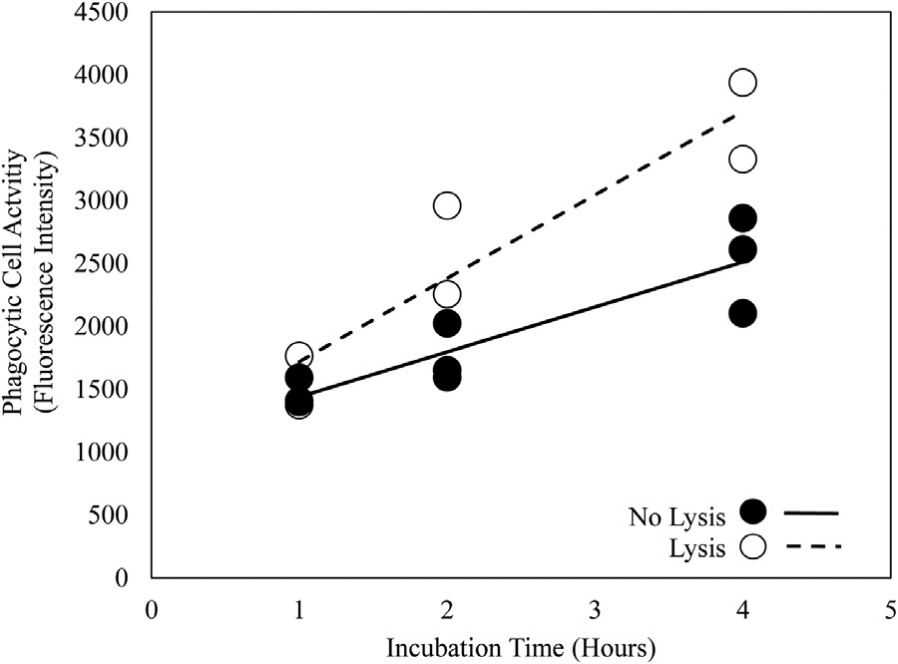
Relationship between phagocytic cell activity and incubation time in untreated fathead minnow kidney cell suspensions and cell suspensions where erythrocytes where lysed (*n* = 2–3).

Differences in the phagocytic cell activity of untreated and erythrocyte-lysed cell suspensions were determined using a two-way ANOVA with lysis and time as factors. The interaction between lysis and time was not statistically significant (Fig. 5, two-way ANOVA, p value = 0.09). However, the main effects of lysis and time were statistically significant (Fig. 5, two-way ANOVA, both p values ≤ 0.01) with cell suspensions incubated for longer periods or subjected to cell lysis exhibiting greater phagocytic cell activity.

The observed increase in phagocytic cell activity observed when erythrocytes were lysed is most likely due to greater relative concentration of leukocytes in the cell suspension and therefore greater numbers of phagocytes relative to other cell types, allowing for greater phagocytic capacity. It is recommended that erythrocytes be lysed if achieving maximum phagocytic activity is critical to a given study design. However, it should be considered that lysing erythrocytes resulted in a ~30–60% reduction in the total number of cells, potentially limiting the number of timepoints or other cellular assays that may be evaluated within a given set of samples given the number of cells required. This dilemma may be mitigated by increasing the amount of pooled tissue, but this would be at the cost of increased animal use. Given that phagocytic cell activity is readily observable in untreated cell suspensions, forgoing erythrocyte lysis may also be acceptable after evaluating the goals of the study at hand. Regardless of treatment decision, it is recommended that multiple timepoints be evaluated to allow for the evaluation of phagocytic cell activity over time.

## Method validation

### Biological variation & repeatability

To assess biological variation and repeatability of the previously described methods, each assay was conducted in three separate trials, each consisting of three samples obtained from different fish for each trial. Conditions were selected based on the results from the method optimization experiments (Superoxide production: 0.5 µg/mL PMA, 60 min incubation, no erythrocyte lysis; Phagocytic cell activity: no erythrocyte lysis, timepoints 1, 2 and 4 h).

**Fig. 6:**
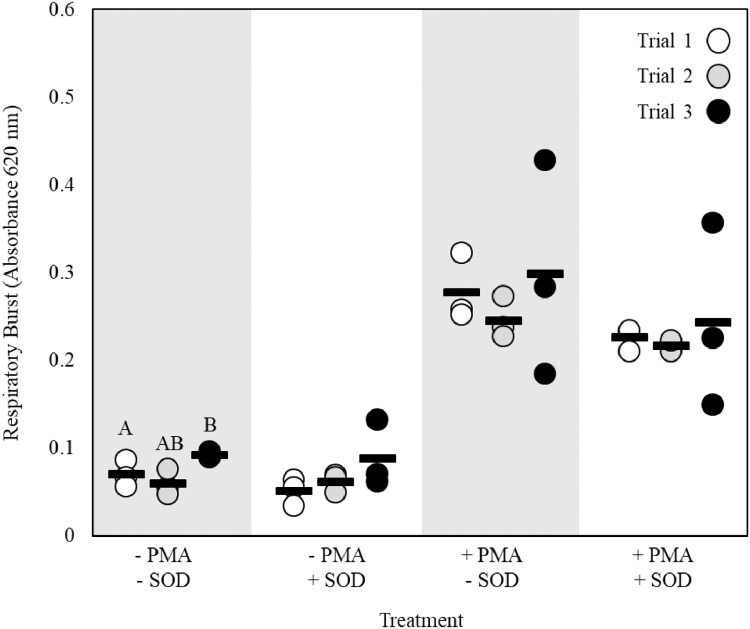
Evaluation of repeatability and variation for respiratory burst in unstimulated or stimulated adult fathead minnow kidney cells across three individual trials (*n* = 3/trial). Different letters indicate significant differences between trials within each treatment. Bars indicate mean. Abbreviations: Phorbol 12-myristate 13-acetate (PMA), Superoxide dismutase (SOD).

For respiratory burst, differences between trials under the same experimental conditions were determined using an ANOVA followed by a Tukey's post-hoc test. In cases of unequal variance, a Kruskal Wallis test was used. No differences were detected between trials when cells were unstimulated and incubated with SOD or stimulated and incubated without SOD (Fig. 6, ANOVA, p values > 0.25). No differences were detected between trials, when cells were stimulated and incubated with SOD (Fig. 6, Kruskal–Wallis, p value = 0.73). However, significant differences were detected between trials when cells were unstimulated and incubated without SOD (Fig. 6, ANOVA, p value = 0.04) where the absorbance of trial 3 was greater than that of trial 2. Though statistically significant, this difference is likely a reflection of inherent differences between individual fish from different trials and may indicate that a larger sample size is required to ensure repeatability due to biological variation in this treatment group.

**Fig. 7:**
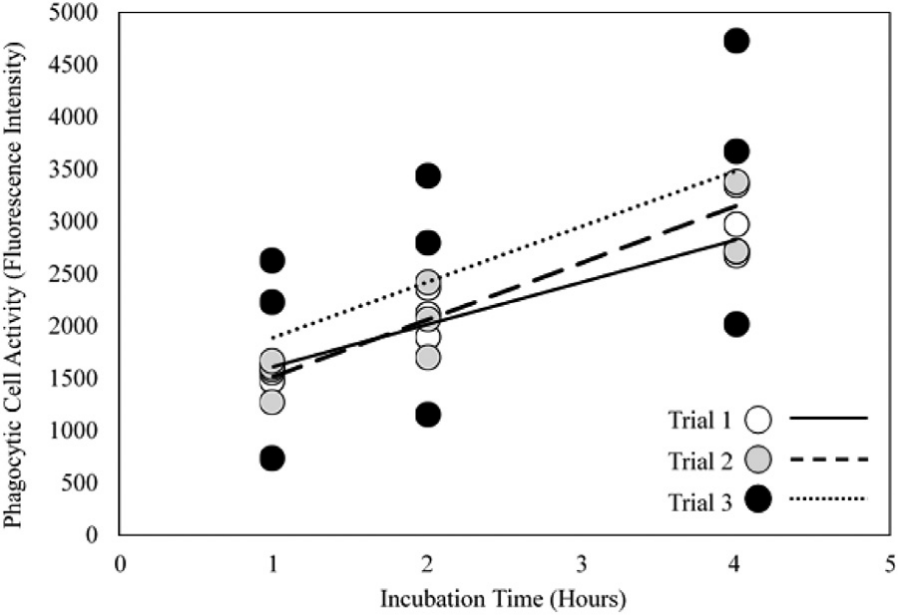
Evaluation of repeatability and variation for phagocytic cell activity in adult fathead minnow kidney cells across three individual trials (*n* = 3/trial).

For phagocytic cell activity, differences between trials were determined using two-way ANOVA with trial and time as the factors. Only time had a statistically significant effect (Fig. 7, two-way ANOVA, p value < 0.01) whereas no differences were detected between trials or the interaction between time and trial (Fig. 7, two-way ANOVA, both p values ≥ 0.39).

### Recommended sample sizes

To determine the sample sizes required for each of these assays, a series of power analyses was conducted using the measured variances of the present study. Specifically, the following formula (which assumes α = 0.05 and β = 0.2 and where n is the estimated sample size, σ is the standard deviation, and D is the absolute value of the predetermined difference between the means (i.e., 20–50% difference between means)) was used [24]:

n≈16(σ/D)2

For each assay, the average standard deviation of the three trials was utilized to estimate sample size. Values for respiratory burst were calculated based on the analysis of unstimulated and stimulated cells in the absence of SOD, and values for phagocytic cell activity were calculated based on the analysis of a single time point (Table 2).

**Table 2:** Sample sizes per group required to detect predicted differences between two means. Values for respiratory burst calculated based on the analysis of unstimulated and stimulated cells in the absence of SOD. Values for phagocytic cell activity calculated based on the analysis of a single timepoint.

		Difference Between Means			
		20%	30%	40%	50%
Respiratory Burst	Unstimulated	9	4	2	2
	Stimulated	20	9	5	3
Phagocytic Cell Activity	1 h	27	12	7	4
	2 h	28	13	7	5
	4 h	16	7	4	3

The estimated sample sizes required for the respiratory burst assay ranged from 2 to 20 samples per group, depending on the predicted differences in the means (Table 2). While the sample sizes required to detect a 20 or even 30% change are relatively large, it should be noted that the coefficients of variation (CVs) between values are comparable to studies utilizing similar methods. Specifically, the mean CVs of unstimulated and stimulated cells were 15 and 23%, respectively, whereas the reported CVs of previous studies were estimated to range from 10 to 60% [3,[25], [26], [27]. This suggests that potentially high levels of variation between samples are possibly inherent to this assay. Researchers should plan accordingly by possibly increasing sample size, particularly when subtle differences between means are predicted.

The estimated sample sizes required for the phagocytic cell activity assay ranged from 3 to 28 samples per group (Table 2). However, it should be noted that sample sizes were estimated based on the assessment of a single timepoint. Therefore, as previously mentioned, it is recommended that multiple timepoints be utilized to gain insight into changes in phagocytic cell activity over time. Should resource or experimental limitations dictate that only a single timepoint be assessed, it is recommended that phagocytic cell activity be evaluated following 4 h of incubation as the required sample sizes are the smallest (Table 2).

### Conclusions

In summary, several assay parameters were experimentally evaluated in an effort to optimize respiratory burst and phagocytic cell activity assays for use with fathead minnow kidney cells. Results of these efforts were utilized to develop the protocols presented here, which appear alongside detailed protocols for kidney tissue extraction and the generation of cell suspensions for use in each assay. Future applications are encouraged to customize and optimize each assay to achieve study objectives or test specific hypotheses. For instance, targets other than *E. coli* may be used to induce phagocytosis, assuming that trypan blue quenches their fluorescence, and the inclusion of positive controls may allow for high-throughput screening applications.

## Declaration of Competing Interest

The authors declare that they have no known competing financial interests or personal relationships that could have appeared to influence the work reported in this paper.

## Appendix Supplementary materials

Image, application 1

Image, application 2

Image, application 3

Image, application 4

Image, application 5

Image, application 6

Image, application 7
