# Supplementary material for: A practical guide for assessing respiratory burst and phagocytic cell activity in the fathead minnow, an emerging model for immunotoxicity
Source: MethodsX. 2020 Jul 10;7:100992. doi: 10.1016/j.mex.2020.100992 (PMC7369328; doi:10.1016/j.mex.2020.100992)
Supplement: Supplementary file 1 [file mmc1.docx]

**Kidney Cell Isolation Standard Operating Procedures**

**Materials**

Tricaine mesylate (MS-222) (0.3 g/L – lethal dose) buffered sodium bicarbonate to ensure a pH consistent with that of the water that fish have been maintained in

General dissection supplies (e.g., forceps, scissors, pins, scalpel, dissection pan)

Heparinized capillary tubes

70% EtOH (preferably in spray bottle)

100 – 250 mL beaker for 70% EtOH

Paper towels

Microscale

Sterile 1.5 mL Eppendorf tubes

Sterile cell media (supplemented Leibovitz’s L-15 cell media (catalog #L5520, Sigma Aldrich)); Fetal Bovine Serum 5% (catalog #F4135, Sigma Aldrich), Penicillin/Streptomycin 1% (catalog #P4333, Sigma Aldrich), 1.5 M HEPES 1% (catalog #H4034, Sigma Aldrich), L-Glutamine 0.5% (catalog #G7513, Sigma Aldrich) – stored at 4°C

Cooler filled with ice

Sterile 1 mL syringes

Paper clip

Sterile glass wool

Plastic pestles

Sterile 5mL screw cap tubes

Centrifuge with adaptors for 5mL tubes

Trypan blue (0.4%) (catalog #76180-676, VWR)

Hemocytometer

Microscope

Tissue Culture Treated 96 Well Flat Bottom Plate (part #667196, Dot Scientific Inc.)

Set of micropipettes and sterile tips

Laminar flow hood

Incubator set at 30°C (CO_2_ not required due to use of L-15 media)

**Procedure**

*Preparation of Materials*

**Note**: Complete the following under sterile conditions in a laminar flow hood.

1. Loosely pack syringes with sterilized glass wool using forceps and unwound paper clip previously doused in 70% EtOH.

2. Pipette 100 μL of previously prepared cell media into sterile 1.5 mL Eppendorf tubes. Fill one tube per sample. Label tubes according to sample number and place on ice outside hood.

3. Label 2 sets of sterile 5 mL screw cap tubes according to sample number.

4. Pipette 90 μL of cell media into 1 set of 1.5 mL Eppendorf tubes and label according to sample number and “1:10”.

*Kidney Tissue Collection*

**Note**: If possible, conduct dissections under sterile conditions in a laminar flow hood. Dissections may be completed outside of a laminar flow hood, but care must be taken to work quickly and decisively to minimize the risk of contamination of tissues and subsequent cell suspensions.

1. Euthanize fish via immersion in a lethal dose of buffered MS-222 (0.3 g/L).

2. Following the cessation of operculum movement, remove fish from MS-222 solution, gently dry on a paper towel and measure total mass.

3. Immediately sever the caudal fin using a scalpel and collect blood in a heparinized capillary tube. Remove as much blood as possible to reduce the numbers of peripheral erythrocytes in the final cell suspension.

4. Use a spray bottle to douse the outside of the fish with 70% EtOH. Dip dissection tools in 70% EtOH and dab dry on a clean paper towel. Begin the dissection normally, taking care not to puncture the intestines. It is recommended to remove viscera up and out to the side of the fish to allow for the easy removal of the kidney tissue.

5. Immediately before removing the kidney tissue, place the Eppendorf tube previously filled with 100 µL of cell media on the microscale and zero. Dip forceps in 70% EtOH and completely dry on a clean paper towel or Kim wipe. Residual ethanol on the forceps will desiccate cells. Remove all kidney tissue from the body cavity and place into pre-tared Eppendorf tube and return to scale. Record kidney tissue mass.

6. Clean dissection pan and dissection tools with 70% EtOH and repeat steps 1-5 for each fish, pooling the appropriate number of tissues together per Eppendorf tube. Return Eppendorf tubes to ice after placing tissues in tube. To determine the number of fish to pool per sample for the desired number of cells for subsequent assays, refer to Figure 1.

Figure 1. Regression analysis between total body mass and cell yield (n = 9) of adult male fathead minnows. Data in panel may be used to predict kidney cell yield from body mass. Dashed lines represents minimum cell yield required to perform both respiratory burst and phagocytic cell assays as described.

*Creation of Cell Suspension*

**Note**: Complete the following under sterile conditions in a laminar flow hood.

1. Once all tissues have been dissected, return to the laminar flow hood and gently homogenize each sample with a sterile plastic pestle. Use a new plastic pestle for each sample.

2. Add 1000 μL of cell media to each sample, rinsing the plastic pestle over the tube in the process. Gently pipette the homogenate up and down ~10-15 times using a 100-1000 μL pipette.

3. To remove remaining large pieces of tissue, remove the plunger and cap of a syringe previously packed with glass wool and hold the syringe over the previously labeled 5mL screw cap tube in case of dripping. Pipette the homogenate into the syringe until full. Gently push the plunger back into the syringe to gently filter the homogenate into the 5 mL screw cap tube.

4. After all samples have been filtered, centrifuge samples for 10 min at ~180 g at room temperature.

5. Remove and dispose of supernatant being careful not to disturb the cell pellet at the bottom of the tube. Add 1 mL of fresh cell media (room temperature), gently pipetting up and down to resuspend the pellet.

6. Repeat steps 4 and 5 for a total of 2 washes.

7. After washing the cells twice, resuspend the cells in 1 mL of fresh media. Gently pipette up and down ~10-15 times to mix. Immediately transfer (cells settle quickly!) 10 μL of cell suspension to the Eppendorf tube containing 90 μL of media and labeled “1:10”.

8. Return all solutions containing cells to ice.

*Determination of Cell Viability and Concentration via Hemocytometer*

**Note**: Steps 1 and 2 may be performed outside of the laminar flow hood. All other steps should be performed inside a laminar flow hood.

1. For each sample, use a 20-200 μL pipette to gently mix the 1:10 dilution of each cell suspension and transfer 10 μL to a new 1.5 mL Eppendorf tube containing 10 μL of trypan blue and mix by gently pipetting up and down. Apply 10 μL of the mixture to the hemocytometer and allow cells to settle for ~30-60 seconds.

2. Count live and dead cells in 1-4 squares on the hemocytometer grid (Figure 2). Average counts together and multiply by the dilution factor (10, if performing a 1:10 dilution), the dilution factor of trypan blue (2) and then by 104 to determine the concentration of cells (ex. mean number of cells in one square x dilution factor x dilution factor of trypan blue x 104 = concentration of cell suspension (cells/mL)). Determine the percentage of live and dead cells to ensure adequate viability (≥ 80%).

3. Adjust the number of live cells to the required concentration for the desired assay by adding the appropriate volume of cell media. Refer to the Cell Suspension Dilution Sheet in the Supplemental Materials for calculating dilutions. For superoxide production and phagocytic activity, cells should be adjusted to 6 x 106 cells/mL and 100 μL is added to each well. A minimum volume of 2600 μL is required per sample to conduct both assays (1300 μL each). When filling wells, be sure to mix cell suspensions often via gentle pipetting. Include blank wells for each treatment with only cell media and no cell suspension. Refer to the Respiratory Burst Plate or Phagocytic Cell Activity Plate in the Supplemental Materials for examples of suggested plate set ups.

4. After cells have been plated, place plate in a 30°C humidified incubator to recover overnight. The injection of CO2 is unnecessary due to the use of Leibovitz’s L-15 cell media.


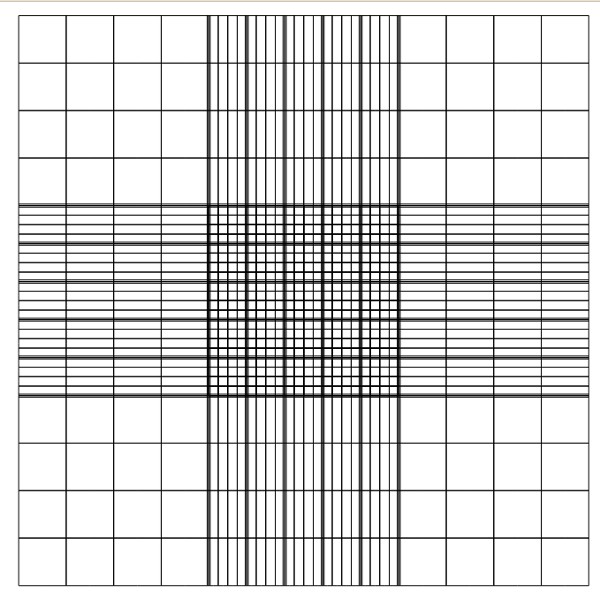


Figure 2. Example of hemocytometer grid. Circled area indicates one “square.”
