# Supplementary material for: A practical guide for assessing respiratory burst and phagocytic cell activity in the fathead minnow, an emerging model for immunotoxicity
Source: MethodsX. 2020 Jul 10;7:100992. doi: 10.1016/j.mex.2020.100992 (PMC7369328; doi:10.1016/j.mex.2020.100992)
Supplement: Supplementary file 3 [file mmc3.docx]

**Respiratory Burst Standard Operating Procedures**

**Materials**

Dissecting microscope

Nitroblue tetrazolium (NBT) (catalog #N6639, Sigma Aldrich) Stock Solution (2.5 mg/mL) prepared in Hank’s Balanced Salt

Solution (HBSS) – To be prepared fresh for each assay

Bovine superoxide dismutase (SOD) (catalog #S5395, Sigma Aldrich) (Stock Solution (300 μg/mL) prepared in HBSS

– stored at -20°C

– Note: Consider the use of membrane-permeable SOD; see section titled, Respiratory Burst: Erythrocyte Lysis and Superoxide Dismutase

Phorbol 12-myristate 13-acetate (PMA) (catalog #76102-732, VWR) 1 mg/mL stock solution prepared in DMSO; Working Solution (100 μg/mL) prepared in HBSS

– stored at -20°C

– light sensitive

Sterile cell media (supplemented Leibovitz’s L-15 cell media (catalog #L5520, Sigma Aldrich)); Fetal Bovine Serum 5% (catalog #F4135, Sigma Aldrich), Penicillin/Streptomycin 1% (catalog #P4333, Sigma Aldrich), 1.5 M HEPES 1% (catalog #H4034, Sigma Aldrich), L-Glutamine 0.5% (catalog #G7513, Sigma Aldrich) – stored at 4°C

70% MeOH

2 M KOH

Dimethyl sulfoxide (DMSO)

Reagent reservoirs

Multichannel pipette

Set of micropipettes and sterile tips

Standard plate reader (absorbance)

Laminar flow hood

Incubator set at 30°C

**Procedure**

**Note**: Complete the following under sterile conditions in a laminar flow hood.

1. After allowing the cells to recover overnight, prepare the following reaction mixes according to Table 1. Refer to the Respiratory Burst Reaction Mix Sheet in the Supplemental Materials for calculating volumes of each component.

2. Gently remove cells from the incubator and observe under a microscope to check the health of the cells. There should be no signs of contamination or cell death.

3. Using a regular or multichannel pipette, remove 50 μL of media from the top of each well. Cells should have settled to the bottom and therefore not be removed from the plate, but if you are at all uncomfortable, it is suggested that a regular pipette is used.

4. After vigorously vortexing, add 50 μL of each reaction mixture in triplicate to each sample. Each sample should receive each reaction mix in triplicate. See Respiratory Burst Plate for further clarification.

7. Immediately after adding the appropriate reaction mixes, return the plate to the incubator for 1 hour.

8. Following incubation, use a multichannel pipette and a reagent reservoir to add 100 μL 70% MeOH to each well.

9. Continuing to use a multichannel pipette (but changing pipette tips each time) immediately remove all solution from the wells and wash each well with an additional 100 μL 70% MeOH 2 more times. After the second wash, remove any remaining solution and allow the plate to air dry at room temperature. Place the plate under a sample box lid without the plate lid on to avoid contaminants falling into the wells.

10. After the plate is completely dry (~20-30 min), use a multichannel pipette to add 120 μL of 2M KOH to each well followed by 140 μL of DMSO and mix thoroughly by pipetting up and down (~10-15 times) to dissolve formazan.

11. Immediately measure the absorbance of the solution on a standard plate reader at 620 nm. Subtract mean values for blank wells from mean values of experimental wells receiving the same reaction mix.

Table 1. Stock solution and final well concentrations of superoxide production reaction mix reagents. The symbol “+” indicates the presence of the regent in the given reaction mix. Refer to the Superoxide Reaction Mix Sheet in the Supplemental Materials for calculating volumes of each component. Note: Nitroblue tetrazolium stock solution is to be made fresh for each assay.

| **Reagent** | **Stock Solution Concentration** | **Final Well Concentration** | **Reaction Mix 1** | **Reaction Mix 2** | **Reaction Mix 3** | **Reaction Mix 4** |
| --- | --- | --- | --- | --- | --- | --- |
| Nitroblue Tetrazolium (NBT) | 2.5 mg/mL | 0.8 mg/mL | + | + | + | + |
| phorbol 12-myristate 13-acetate (PMA) | 100 µg/mL | 0.5 µg/mL |  |  | + | + |
| Superoxide Dismutase (SOD) | 300 µg/mL | 30 µg/mL |  | + |  | + |
