# Supplementary material for: A practical guide for assessing respiratory burst and phagocytic cell activity in the fathead minnow, an emerging model for immunotoxicity
Source: MethodsX. 2020 Jul 10;7:100992. doi: 10.1016/j.mex.2020.100992 (PMC7369328; doi:10.1016/j.mex.2020.100992)
Supplement: Supplementary file 6 [file mmc6.docx]

**Phagocytic Cell Activity Standard Operating Procedures**

**Materials**

Dissecting microscope

Fluorescein (FITC) conjugated *Escherichia coli* K-12 BioParticles (0.5 mg/mL) prepared in HBSS and 2 mM sodium azide (catalog #E2861, ThermoFisher Scientific)

– stored at 4°C

– protected from light

Vortex

Trypan blue (0.4%) (catalog #76180-676, VWR)

Aluminum foil

Set of micropipettes and sterile tips

Centrifuge for 96 well plates

Standard plate reader (fluorescence)

Incubator set at 30°C

Laminar flow hood

**Procedure**

**Note**: Complete the following under sterile conditions in a laminar flow hood.

1. Gently remove cells from the incubator and observe under a microscope to check the health of the cells. There should be no signs of contamination or cell death.

**Note**: Plan ahead in the following steps to reduce the time cells are outside of the incubator as much as possible. Phagocytosis occurs rapidly. Therefore, any preliminary steps taken to increase efficiency while taking fluorescence measurements will be beneficial (i.e., plate reader is on and correct acquisition protocol has been previously programmed).

2. Acquire the FITC conjugated E. coli bioparticles (0.5 mg/mL), vortex and add 40 µL to all sample and blank wells. This equates to a 1:10 cell to particle ratio based on manufacturer specifications for the number of particles per mg.

3. Immediately add 50 µL to the 0-hour timepoint wells and centrifuge the plate at 500 rpm for 5min to bring particles to the bottom of each well and facilitate particle to cell interaction.

4. Immediately measure the fluorescence intensity of the wells corresponding to the 0-hour timepoint using the standard plate reader (excitation filter: λ = 488 nm, emission filter: λ = 518 nm).

5. Return the plate to the incubator and begin timer for the next timepoint. Loosely cover plate in foil to avoid exposure to light.

6. At each timepoint, remove the plate from the incubator, add 50 µL of trypan blue to the wells corresponding to the given timepoint and measure the fluorescence intensity as previously described. There is no need to centrifuge the plate each time. Repeat until all chosen timepoints have been completed.

7. To determine the amount of phagocytic cell activity over time, subtract the mean fluorescence intensity values at 0 h from the mean fluorescence intensity values at each subsequent timepoint to eliminate background fluorescence in each sample.
